# Growth hormone receptor promotes osteosarcoma cell growth and metastases

**DOI:** 10.1002/2211-5463.12761

**Published:** 2019-12-18

**Authors:** Mo Cheng, Wending Huang, Weiluo Cai, Meng Fang, Yong Chen, Chunmeng Wang, Wangjun Yan

**Affiliations:** ^1^ Department of Musculoskeletal Surgery Fudan University Shanghai Cancer Center China

**Keywords:** growth hormone receptor, metastasis, osteosarcoma, proliferation, tumor growth

## Abstract

Osteosarcoma (OS) is the primary bone malignancy in children and adolescents, with a high incidence of lung metastasis and poor prognosis. Here, we report that growth hormone receptor (GHR) is overexpressed in OS samples compared with osteofibrous dysplasia. We subsequently demonstrated that GHR knockdown inhibited colony formation, promoted cell apoptosis and decreased the number of cells at G2/M phase in 143B and U2OS cells. Furthermore, knockdown of GHR inhibited tumor growth *in vivo*. Together, these findings indicate that GHR modulates cell proliferation and metastasis through the phosphoinositide 3‐kinase/AKT signaling pathway and may be suitable for use as a putative biomarker of OS.

## Graphical abstract

Here, we demonstrate that growth hormone receptor knockdown inhibits colony formation, promotes cell apoptosis and decreases the number of cells at G2/M phase in 143B and U2OS cells. Furthermore, knockdown of growth hormone receptor inhibits tumor growth *in vivo*.

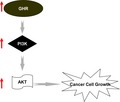

**Abbreviations**
GH: growth hormone
GHR: growth hormone receptor
mTOR: mammalian target of rapamycin
NKX: NK homeobox
OS: osteosarcoma
PI3K: phosphoinositide 3‐kinase
SOCS: cytokine signaling

Growth hormone receptor (GHR) signaling is important in a range of cellular processes, including aging 1, metabolic processes 2 and nuclear translocation 3. Growth hormone (GH) activates mitogenic signaling through Janus kinase/signal transducers and activators of transcription, mitogen‐activated protein kinase/extracellular signal‐regulated kinase and mammalian target of rapamycin (mTOR) pathways 4. GH is the inducer of P‐45015/S and IGF‐I gene. GHR is widely expressed in GH target cells, is the key regulator of postnatal growth, and holds important actions over metabolic processes, human stem cell function and aging 3, 5. A recent study indicates that mTOR complex‐1 activity is lower in liver, muscle, heart and kidney tissue of global GHR gene‐disrupted mice (GHR^−/−^) 6. In addition, GHR SNP promotes lung cancer progression by impairment of cytokine signaling 2 (SOCS2)‐mediated degradation 7; meanwhile, one review focused on the description of the inhibitors of the GHR/Janus kinase 2/signal transducers and activators of transcription pathway, which also includes SOCS1, SOCS2 and SOCS3 8. Functionally, on the one hand, disruption of the GHR gene (*Ghr* gene) in the liver is characterized by lack of improved insulin sensitivity and severe hepatic steatosis 9; on the other hand, GHR deficiency protects against age‐related NLRP3 inflammasome activation and immune senescence 10. In human melanoma cells, knockdown of GHR promotes sensitivity of chemotherapy 1. In aggregate, GHR plays a key role in tumors.

Osteosarcoma (OS) is the primary bone malignancy cancer in children and adolescents, with a high incidence of lung metastasis and a poor prognosis, and it remains a leading cause of cancer‐related deaths 11, 12, 13. Angiopoietin‐like 4 (ANGPTL4) can enhance osteoclast activity and further promote OS growth in bone 14. In addition, upregulating vascular cell adhesion molecule 1 (VCAM‐1) expression stimulates OS cell migration and induces OS metastasis 15. Dysregulated Livin and PlGF may participate in the pathogenesis of OS 16. Some reported that the genes such as MSTN, CCND2, Lin28B, MEST, HMGA2 and GHR may represent new biomarkers for OS 17. Despite these predictive bookmarks, serine/threonine kinase receptor‐associated protein is upregulated during OS progression, which enhanced OS cell invasion and metastasis 18. GIT1 participated in OS growth, invasion and angiogenesis 19. In contrast, Let7 was significantly underexpressed in OS cell lines; therefore, Let7 inhibits OS growth and lung metastasis by targeting Aurora‐B 20. Analogously, a member of the NAD‐dependent sirtuin family of enzymes, SIRT6, a tumor suppressor, targeted N‐cadherin and inhibited proliferation and invasion of OS cells 21. NK homeobox (NKX) genes, especially *NKX2‐2*, a tumor suppressor for OS, and overexpression of NKX2‐2 decreased the migration, invasion, proliferation and colony formation of OS cells 22. In recent years, some miRNA and long noncoding RNAs were involved in OS progression, such as miR‐34a/cyclin D1 axis, miR‐216b, miR‐423‐5p and long noncoding RNA GHET1 23, 24, 25, 26. However, the involvement of GHR in OS has not been reported.

In this study, we proceeded to investigate the role of GHR in the modulation of OS progression and explored its potential as a biomarker and new strategy for OS therapy.

## Results

### GHR is overexpressed in patients with OS

**Figure 1:**
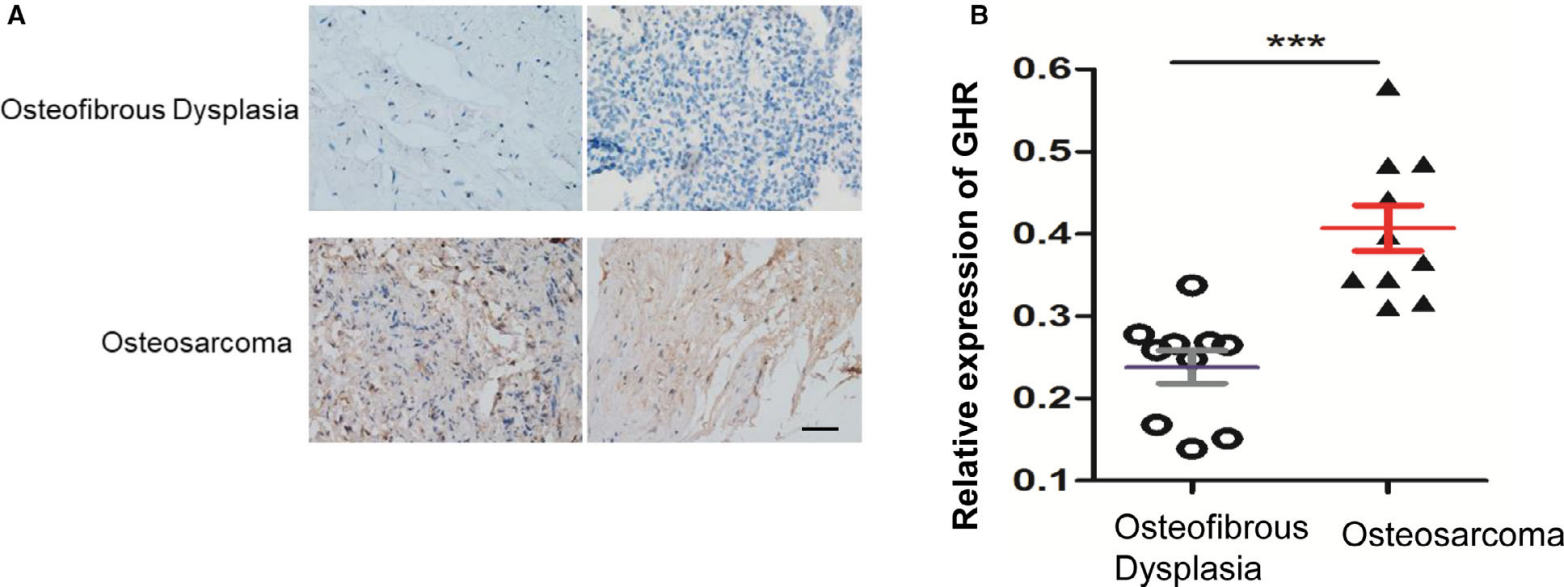
GHR is overexpressed in OS samples. (A) GHR was overexpressed in the tissue of patients with OS. Scale bar, 50 μm (original magnification ×20). (B) Statistical analysis of the expression of GHR in (A). Data represent mean ± SEM (*n* = 10). Data are presented as mean ± SEM; two‐tailed Student’s *t*‐test was used for statistical analysis, ***P < 0.001.

To obtain the new function of GHR in OS, immunohistochemistry results showed that GHR was overexpressed in the tissue of patients with OS, compared with osteofibrous dysplasia (Fig. 1A). Furthermore, statistical analysis experiments confirmed the results (*n* = 10; Fig. 1B). These clinical data suggested a positive link between OS and GHR level.

### Knockdown of GHR inhibits OS cell growth through the phosphoinositide 3‐kinase/AKT pathway

**Figure 2:**
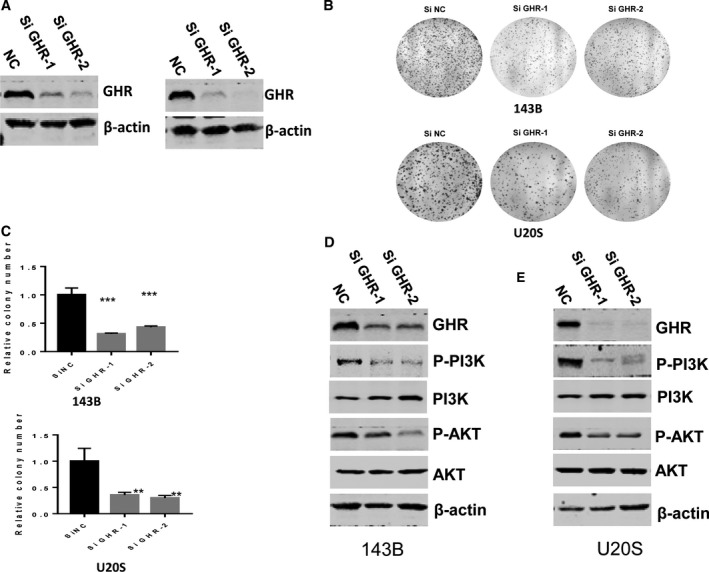
GHR promotes OS cell colony formation and OS tumor growth through the PI3K/AKT pathway. (A) The expression of GHR was detected by western blotting. (B) Control and GHR knockdown 143B and U2OS cells were analyzed by colony formation. (C) Statistical analysis of the colony formation in (B). Data represent mean ± SEM (*n* = 3); two‐tailed Student’s *t*‐test was used for statistical analysis, **P < 0.01, ***P < 0.001 in silenced GHR cells (D, E) Knockdown of GHR by two independent siRNAs inhibited the expression of p‐PI3K/AKT in 143B (D) and U2OS (E) cells.

To investigate the biological function of GHR in OS cells, we transfected siRNA against GHR to 143B and U2OS cell lines, and the data identified the decreased expression of GHR (Fig. 2A). Next, we evaluated the cell growth involved with GHR, and the results showed that knocking down GHR inhibited colony formation of 143B and U2OS (Fig. 2B,C). The phosphoinositide 3‐kinase (PI3K)/AKT/mTOR pathway is frequently activated in various human cancers and has been considered a promising therapeutic target. Therefore, we defined that the expression of phosphorylated (p)‐PI3K and p‐AKT was decreased in silenced GHR cells (Fig. 2D). Our data verified that GHR modulated OS growth through the PI3K/AKT pathway.

### GHR modulates the proliferation of OS cells *in vivo*

**Figure 3:**
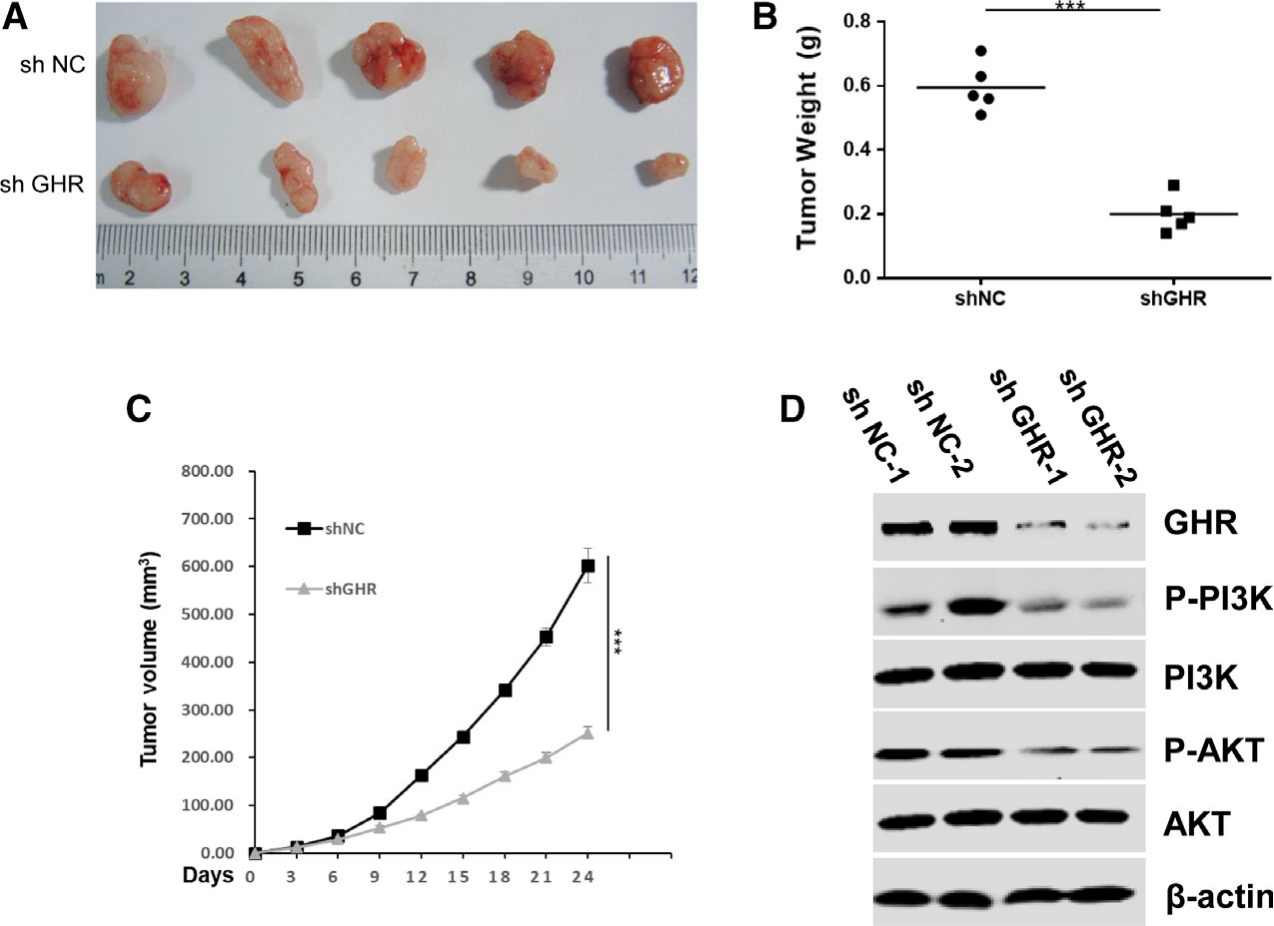
GHR modulates the proliferation of OS cells *in vivo*. (A–C) Control and GHR knockdown U2OS cells were injected into nude mice subcutaneously (2 × 10^6^ cells per mouse); tumor volume and weight were measured at the indicated day. Data represent mean ± SEM (*n* = 5); two‐tailed Student’s *t*‐test was used for statistical analysis, ***P < 0.001. (D) Western blotting analysis of p‐PI3K/AKT for GHR knockdown (sh GHR) or control (sh NC) tumors.

To further verify the function of GHR on tumor growth, we generated stable cells in U2OS cells by transfecting lentivirus expressing shGHR (Knockdown) and shCtrl (Control) plasmid. Then, shGHR and shCtrl U2OS cells (2 × 10^6^ cells) were subcutaneously injected into 4‐ to 6‐week‐old BALB/C (nu/nu) female nude mice (five mice in each group). Notably, the tumor volume and weight were much lower in the shGHR group (Fig. 3A–C). Collectively, these results revealed that knockdown of GHR could inhibit OS cell colony formation and reduce tumor growth. More importantly, western blotting analysis of p‐PI3K/AKT in tumors also showed decreased p‐PI3K and p‐AKT levels in shGHR tumors (Fig. 3D).

### Knockdown of GHR induces G2/M phase arrest and stimulates cell apoptosis

**Figure 4:**
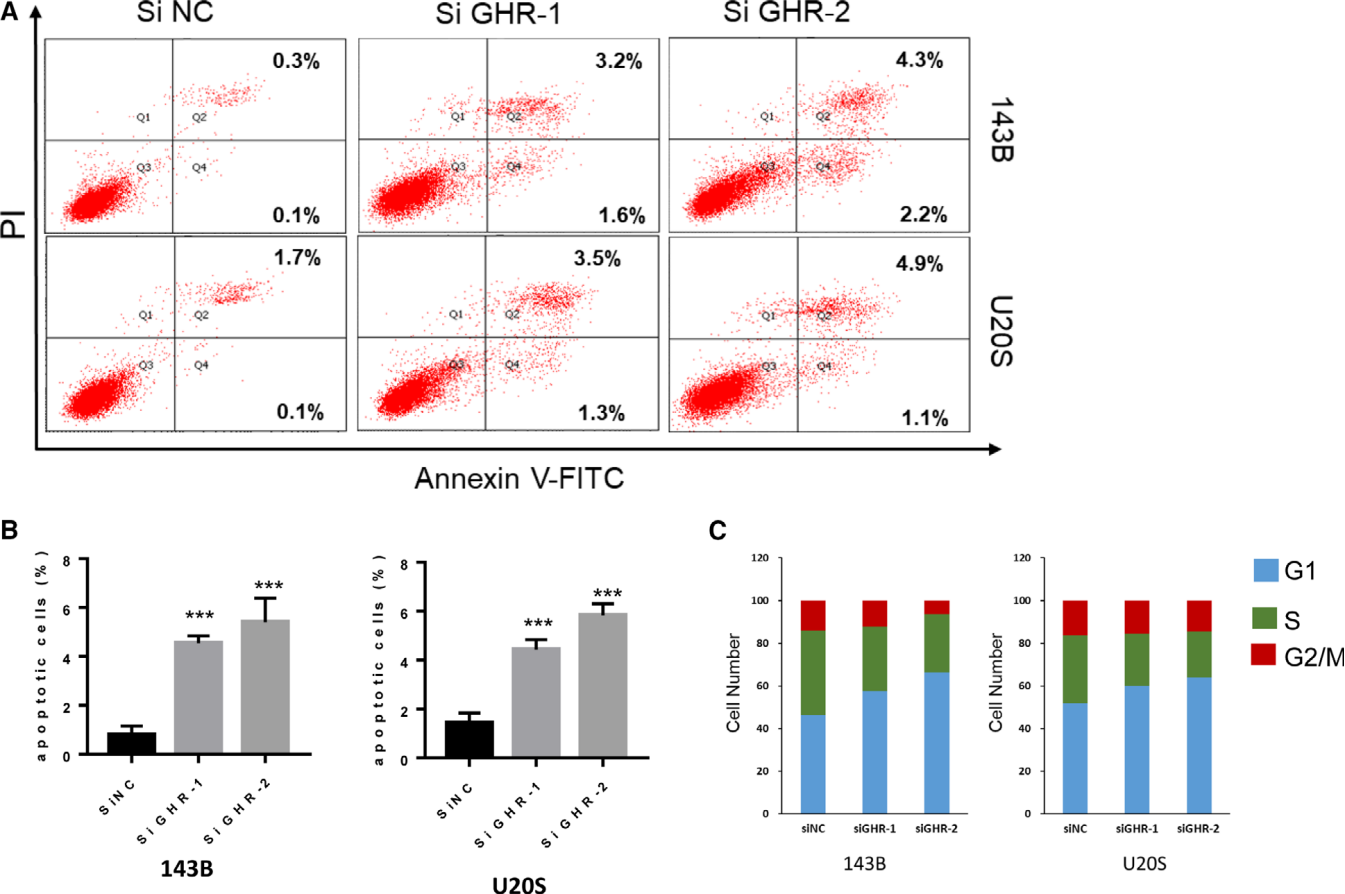
GHR enhances G2/M phase transition and inhibits cell apoptosis. (A, B) The cell proliferation was analyzed by PI and annexin V staining in control and GHR knockdown 143B and U2OS cells. Data represent mean ± SEM (*n* = 3); two‐tailed Student’s *t*‐test was used for statistical analysis, ***P < 0.001. (C) Control and GHR knockdown 143B and U2OS cell cycle transition by flow cytometry.

Because GHR is identified as a potent proliferation inducer and defective growth is usually associated with dysregulated apoptosis, we therefore measured the apoptosis in the GHR knockdown cells. We used the annexin V and propidium iodide (PI) double‐staining kit to quantify GHR‐regulated cell apoptosis. After silencing GHR, annexin V/PI staining showed an increased rate of cell apoptosis in different OS cells (Fig. 4A,B). Furthermore, cell cycle changes were analyzed by fluorescence‐activated cell sorting assay. Interestingly, we observed that the cell numbers of G1/S phase decreased in siGHR 143B and U2OS cells (Fig. 4C). Thus, GHR promoted OS cell proliferation and tumor growth by inhibiting cell cycle arrest and suppressing cell apoptosis.

### Knockdown of GHR inhibits OS cell migration

**Figure 5:**
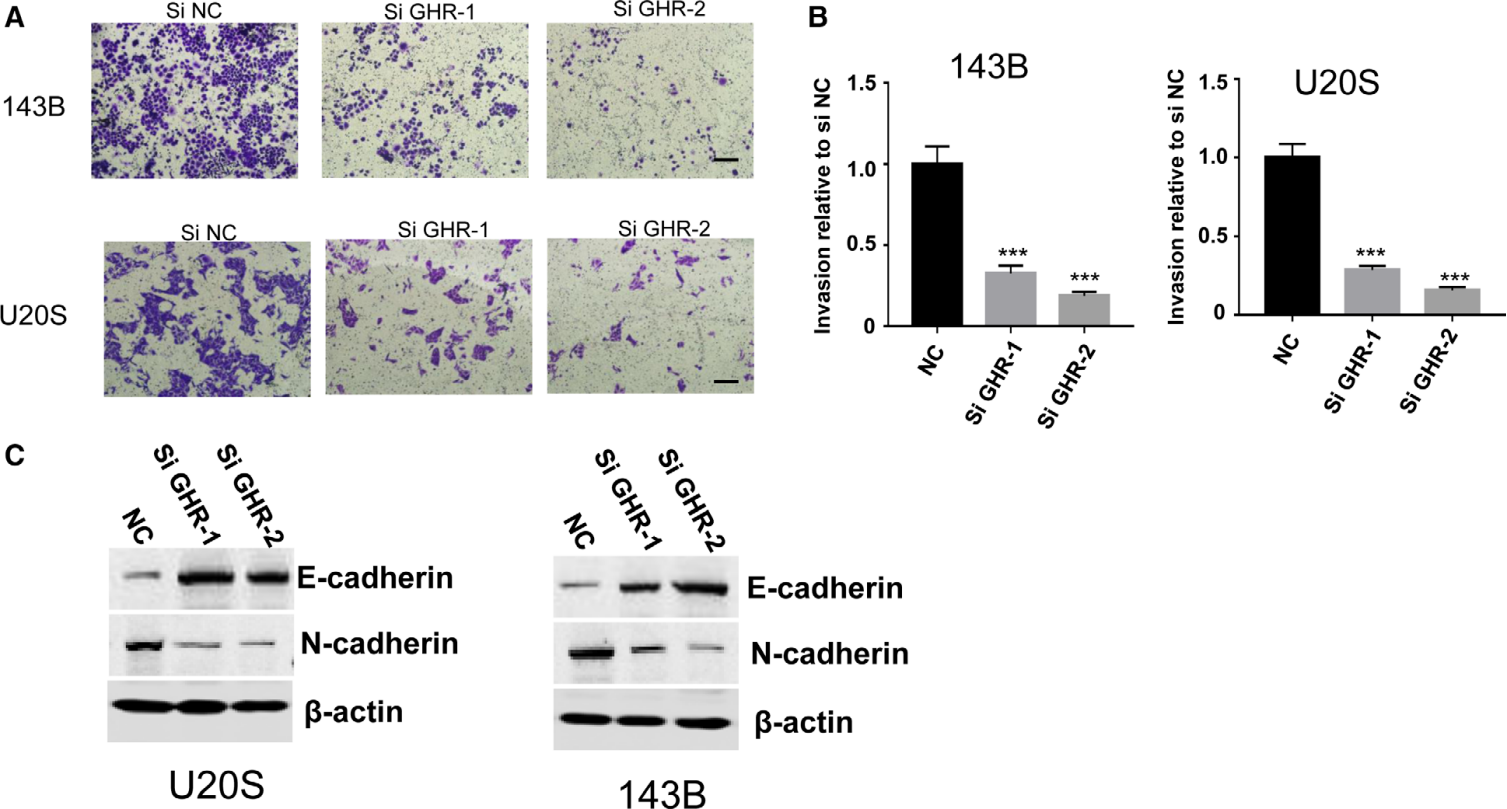
GHR promotes OS cell migration. (A, B) Knockdown of GHR by two independent siRNAs inhibited invasion capacity of 143B and U2OS cells as determined by Transwell assays. Scale bars: 50 μm (original magnification ×10). Data are presented as mean ± SD; two‐tailed Student’s *t*‐test was used for statistical analysis, ***P < 0.001. (C) Knockdown GHR in U2OS and 143B cells, and expression of EMT markers E‐cadherin and N‐cadherin were determined by western blot.

To explore the impact of GHR on cell migration, we used Transwell assays, which demonstrated that GHR normal cells are more invasive than silenced 143B and U2OS cells (Fig. 5A). Then, the statistical analysis results also demonstrated decreased migration after GHR knockdown (Fig. 5B). To gain further insight into the role of GHR in epithelial–mesenchymal transition (EMT), we tested the change of EMT markers (E‐cadherin and N‐cadherin). Knockdown of GHR in 143B or U2OS cells showed increased E‐cadherin and decreased N‐cadherin expression (Fig. 5C). Taken together, our results indicated that GHR promoted cell migration by regulating the expression of EMT markers such as E‐cadherin and N‐cadherin.

## Discussion

In this study, we uncovered the biological function of GHR in OS. Proliferation and metastasis is a critical step in developing OS; however, the specific mechanism is unknown. In this study, novel findings are: (a) GHR was overexpressed in the tissue of patients with OS compared with osteofibrous dysplasia (Fig. 1); (b) GHR promoted OS cell colony formation and OS tumor growth through the PI3K/AKT pathway (Fig. 2); (c) GHR modulated the proliferation of OS cells *in vivo* (Fig. 3); (d) GHR enhanced G2/M phase transition and inhibited cell apoptosis; and (e) GHR promoted OS cell migration. Together, these data indicate that elevated GHR expression could play a role in the development and progression of OS.

It has been documented that OS is a highly metastatic tumor, for which there was no chemotherapy 27. The functional significance of GHR expression and mutation is still unexplored. Thus, analysis of the GHR biological function and detecting the mechanism of metastasis are urgent in OS. GHR was overexpressed, and knockdown of GHR significantly decreased the proliferation and colony formation of 143B and U2OS cells. This finding is consistent with xenograft tumors in nude mice, in which GHR knockdown inhibited tumor growth. It was reported that GHR can directly influence bone cells and played an important role in osteoblasts 28. We further investigated the mechanism of GHR‐promoted cell proliferation. The role of GHR in the cell cycle has been identified: knocking down GHR caused G2/M phase arrest, with a corresponding increase in the population of cells entering G1 and S phases. Previous study had shown that MALAT1 inhibited OS progression via regulating the miR‐34a/cyclin D1 axis. It had been reported that MALAT1 functioned as a ceRNA to suppress miR‐34a expression and in turn upregulated CCND1 in OS cells 26. Consistent with this report, we also found that knockdown of GHR could aggregate the cell apoptosis in 143B and U2OS, suggesting that silencing GHR inhibited cell proliferation and tumor growth via increased cell apoptosis rate and decreased cell numbers of G2/M phase.

We further investigated the role of GHR in OS metastasis. Silencing GHR could result in decreased migration when GHR expression was suppressed. Then, we explored the mechanism of the declining migration. Surprisingly, we found decreased E‐cadherin expression. This finding was consistent with patients with OS. Osteogenesis, like ANGPTL4, promotes OS cell proliferation and migration, and stimulates osteoclastogenesis 14. Our data suggest that GHR promoted migration via the EMT‐dependent pathway; meanwhile, the involvement of GHR in cell migration may lead to osteofibrous dysplasia.

Furthermore, we evaluated the potential regulatory pathway through which GHR modulates the proliferation and migration progression of OS cells. It has been reported that OS metastasis via the αvβ3 integrin/FAK/PI3K/Akt/nuclear factor‐κB signaling pathway and PI3K/AKT signaling pathway might promote OS cell growth. In addition, AKT signaling may be responsible for OS cell proliferation 12, 29. Thus, our findings revealed that the PI3K/AKT pathway is necessary for OS cell proliferation and migration. However, the other mechanism of migration remains to be elucidated.

In summary, we demonstrate that GHR is overexpressed in OS patients. Our finding suggests that GHR modulates OS cell proliferation and metastasis through the PI3K/AKT pathway and EMT. Herein, we speculate that GHR may be the biomarker of patients with OS with osteofibrous dysplasia and provide a promising avenue for prognostic and therapeutic strategies to improve patient outcomes.

## Material and methods

### Cell culture

All of the human OS cells 143B and U2OS were obtained from American Type Culture Collection (Manassas, VA, USA). U2OS cells were cultured with 15% FBS (Gibco, Carlsbad, CA, USA) and Dulbecco’s modified Eagle’s medium.

### Western blot analysis

Cells were collected with PBS, 0.25% Trypsin (Gibco), Dulbecco’s modified Eagle’s medium containing 10% FBS and lysate with lysis buffer containing a cocktail of protease inhibitors. Next, waiting for 30 min on ice, then centrifuging at 10 000 ***g*** for 15 min at 4 °C, the supernatant was collected and then made an equal quality to every protein sample for western blot. These proteins were electrophoresed (80 V in spacer gel, 100 V in separation gel) in 10% SDS/PAGE gels and then transferred (260 mA) to nitrocellulose membranes (Millipore, Bedford, MI, USA). The membranes were blocked with 5% skim milk at room temperature for 1 h and then washed three times with Tris‐buffered saline with 0.1% Tween 20 for 10 min each time. The membrane was incubated with primary antibody at 4 °C overnight. Then it was incubated with the second antibody, and the protein was recognized by chemiluminescence (Amersham, Marlborough, MA, USA). Western blots were visualized using a Bio‐Rad XRS autoimager (Bio‐Rad, Hercules, CA, USA). Relative intensities of bands were quantified by software image lab 4.0.1 (Bio‐Rad) normalized to actin. The used antibodies were as follows: actin (Sigma‐Aldrich, St. Louis, MO, USA), GHR (Proteintech, Shanghai, China), p‐PI3K (Cell Signaling, Shanghai, China), p‐AKT (Cell Signaling), PI3K (Cell Signaling), AKT (Cell Signaling), Goat anti‐Rabbit (Thermo, Danvers, MA, USA) and Goat anti‐Mouse (Thermo).

### siRNA and plasmids transfection

Transient transfection was performed by using Lipofectamine 2000 (Invitrogen, Carlsbad, CA, USA) according to the manufacturer’s protocols. The target GHRs of two siRNAs were 5‐GCAACCAGAUCCACCCAUUTT‐3 and 5‐GCACCACGCAAUGCAGAUATT‐3.

### Cloning formation assay

Cell proliferation was assessed using a Cell Counting Kit‐8 colorimetric assay system (Dojindo Molecular Technologies, Inc., Rockville, MD, USA). Assays were conducted on targeted knockdown (siGHR) cells and control cells at a density of 1 × 10^4^ cells per well. The *A*_450 nm_ was measured according to the manufacturer’s instructions for each time point of the assay.

### Immunohistochemistry assay

Formalin‐fixed OS sections were deparaffinized using xylene and then rehydrated with graded alcohols and, finally, distilled water. After being treated with 3% H_2_O_2_ for 15 min, the slides were treated for antigen retrieval in a microwave oven for 5 min repeated three times and then cooled down to room temperature slowly with PBS. After 30‐min incubation in 10% goat serum, the sections were incubated in proper primary antibodies (GHR, 1 : 100 dilution) overnight at 4 °C. After washing three times with PBS, the sections were incubated in horseradish peroxidase‐conjugated secondary antibodies (1 : 500 dilution), and the subsequent detection was performed using the standard substrate detection of horseradish peroxidase. Then, the sections were stained with hematoxylin and dehydrated in graded alcohols and xylene 30.

### Cell apoptosis assay

Suspension cells were collected by centrifugation (500 ***g*** for 5 min); adherent cells were collected by trypsin digestion without EDTA. The procedure is as follows: wash the cells twice with PBS (centrifugation at 2000 r.p.m. for 5 min) to collect 1–5 × 10^5^ cells; add 500 μL of binding buffer suspension cells; and after adding 5 μL of annexin V–EGFP, add 5 μL of PI and mix.

### Cell metastasis and collective invasion assays

After Transwell chamber preparation, add 100–200 μL of cell suspension to the Transwell chamber; a conventional culture is 12–48 h. To determine cell counts, use an upright microscope for observation and photographing.

### Animal model assay

After collecting and counting U2OS cell numbers, we mixed the equal volume Matrigel (BD, San Jose, CA, USA) with cell resuspension (2 × 10^6^∙100 μL^−1^ in PBS containing 50% Matrigel). All animal studies were carried out in accordance with established institutional guidelines and approved protocols by Fudan University Shanghai Cancer Center. We injected cells to female nud/nud mice (4–6 weeks) subcutaneously. Tumor formation was monitored for 8 weeks. The tumor volumes were determined by Vernier caliper. Tumor volume was calculated by the formula: *V* = 0.5 × *a *× *b* ^2^, where *a* represents tumor length and *b* represents tumor width.

### Clinical tissue samples

Our research was approved by the Ethics Committee of Fudan University Shanghai Cancer Center, and written informed consent was obtained from each patient. A total of 10 OS tissues and 10 osteofibrous dysplasia tissues were collected from patients with OS and osteofibrous dysplasia who underwent spine surgery at the Department of Orthopedic Oncology of Shanghai Cancer Center between 2015 and 2018. The study methodologies conformed to the standards set by the Declaration of Helsinki.

### Database of patients with OS

Datasets in Oncomine (https://www.oncomine.org) were also examined following previous methods.

### Statistical analysis

Differences between the control and experimental samples were assessed using two‐tailed Student’s *t*‐tests. All statistical analyses were performed using graphpad prism software (Graph Pad Software, La Jolla, CA, USA), with a *P* < 0.05 threshold used to assess significance. Each value is reported as the mean ± standard error of the mean.

## Conflict of interest

The authors declare no conflict of interest.

## Author contributions

MC and WY participated in the design of the study and performed the measurements and the statistical analysis. MC, WH, WC, MF, YC and CW helped in data collection and the interpretation of data. WY wrote the manuscript. All authors read and approved the manuscript.
